# Optimizing the Infrared Photoelectric Detection Performance of Pbs Quantum Dots through Solid-State Ligand Exchange

**DOI:** 10.3390/ma15249058

**Published:** 2022-12-19

**Authors:** Mei Yang, Huan Liu, Shuai Wen, Yuxuan Du, Fei Gao

**Affiliations:** School of Optoelectronic Engineering, Xi’an Technological University, Xi’an 710021, China

**Keywords:** lead sulfide (PbS), quantum dots (QDs), solid-state ligand exchange, photodetectors

## Abstract

Lead sulfide (PbS) quantum dots (QDs) have attracted a great deal of attention in recent decades, due to their value for applications in optoelectronic devices. However, optimizing the performance of optoelectronic devices through ligand engineering has become a major challenge, as the surfactants that surround quantum dots impede the transport of electrons. In this paper, we prepared PbS QD films and photoconductive devices with four different ligands: 1,2-ethylenedithiol (EDT), tetrabutylammonium iodide (TBAI), hexadecyl trimethyl ammonium bromide (CTAB), and sodium sulfide (Na_2_S). A series of characterization studies confirmed that using the appropriate ligands in the solid-state ligand exchange step for thin film fabrication can significantly improve the responsivity. The devices treated with sodium sulfide showed the best sensitivity and a wider detection from 400 nm to 2300 nm, compared to the other ligand-treated devices. The responsivity of the champion device reached 95.6 mA/W under laser illumination at 980 nm, with an intensity of 50 mW/cm^2^.

## 1. Introduction

Commercial infrared (IR) detectors are expensive because of their epitaxial growth methods and complicated integration processes. Their performance is optimized after cooling, further increasing integration complexity, power consumption, and miniaturization [1,2,3]. Quantum dots (QD) are considered ideal candidate material. By controlling the particle size of QD, a wide range of spectral responses from ultraviolet to near infrared (NIR) regions can be achieved. Quantum dots dispersed in solution are not limited by the type of substrate materials, and devices can be prepared by spin coating, spraying, or ink-jet printing technology [4,5].

In recent years, people have developed a particularly strong interest in PbS QDs, and the nanomaterial synthesis method has been developed quite well [6,7]. PbS quantum dots have a very small band gap (0.41 eV), large exciton Bohr radius (18 nm), and high dielectric constant, which makes PbS QDs have wider band gap tunability and higher carrier mobility [8]; they are widely used in photodetectors [9,10], solar cells [11], and light-emitting diodes [12]. 

In QDs-based photodetectors, a major obstacle against high performance is the low mobility of the quantum dot films, primarily due to inefficient charge-hopping transport [13]. The surfaces of QDs typically possess long-chain and electrically insulating organic ligands, which need to be shortened or removed in order to be used in optoelectronic device applications [14,15]. The design of ligand exchange or chemical treatment with shorter molecules to reduce the spacing between the quantum dots, shortening the carrier transport distance between the quantum dots and improving the carrier transport capacity to create more conductive quantum dot films, is one of the major directions of current research in quantum dots [16,17,18].

In the early days, nanocrystalline ligand exchange was mostly substituted with organic molecules such as butylamine or pyridine [19]. Sargent and co-workers created ultra-sensitive photodetectors for visible and infrared wavelengths through the exchange of organic oleic acid ligands with sulfates [20,21]. Metal sulphides have attracted attention due to their excellent physical and chemical properties [22]. An inorganic ligand substitution strategy based on metal–sulfur complexes (MCC) was reported by Talapin et al. [23]. In 2010, they further developed an MCC-based strategy for ligand substitution at the colloidal quantum dot film level to passivate surface defects and enhance electron coupling and charge transport [24]. Tang et al. established an atomic ligand strategy that makes use of monovalent halide anions to enhance electronic transport. They used methanolic solutions of common inorganic salts to immerse PbS quantum dot films and obtained the first fully inorganic quantum dot films passivated by halogen ions [11]. Inorganic ligand substitution has a certain degree of universality and is expected to be extended to most simple inorganic salts for the fabrication of a wide range of functional devices based on quantum dot semiconductor materials. Moreover, in contrast to the large number of studies on solar cells, only a few studies exist on the effect of ligands on infrared photoelectric detectors [25].

In the present study, we investigated the effects of four different ligands, EDT, TBAI, CTAB, and Na_2_S, on the films and the responses of the PbS QD photodevices. The PbS quantum dot films covered with these ligands were investigated using Fourier infrared spectroscopy (FTIR), transmission electron microscopy (TEM), field-emission scanning electron microscopy (SEM), and X-ray photoelectron spectroscopy (XPS). In addition, photoconductive devices containing these ligand-covered PbS QD films as active layers were prepared to compare their photovoltaic properties. Overall, the results showed that Na_2_S proved to be the most suitable ligand for the PbS QDs synthesized in this study, and that it can broaden the range of absorption spectra, complementing the known bandgap tunability strategy for PbS quantum dots.

## 2. Materials and Methods

### 2.1. Synthesis of PbS Quantum Dots

The synthesis of PbS QDs followed the method reported by Hines, with a few modifications [7]. Amounts of 0.45 g of lead oxide (PbO), 1.25 mL of oleic acid (OA), and 18.75 mL of 1-octadecene (ODE) were added into the three-necked flask. After vacuuming, the solution was heated to 150 degrees Celsius with stirring under a nitrogen atmosphere and maintained for one hour. The solution then became transparent and gold-yellow. The pre-made sulfur precursor (consisting of 10 mL of octadecene mixed with 210 microliters of hexamethyldisilathiane (TMS_2_S)) was then rapidly injected, and the solution in the vial rapidly turned black, indicating the formation of nanocrystals. The reaction was terminated with an ice-water bath after five minutes of reaction. Acetone was added to centrifuge the PbS QDs, and then the quantum dots were purified by toluene/acetone dispersion/precipitation, which was repeated three times and finally dispersed in toluene.

### 2.2. Device Fabrication

A gold cross-finger electrode with a channel length of 5 µm was used to make the photodetector. The active layer of the device was formed by a continuous layer-by-layer spin coating technique, and all processing was carried out in air. In the case of the monolayer, a solution of PbS quantum dots (30 mg/mL) in toluene was added dropwise to the substrate and immediately spun at 2500 rpm for 30 s. To perform the ligand exchange process, the ligand solution was dropped onto the PbS QD film and allowed to stand for 1 min; then, the coating was rotated for 30 s. The resulting PbS film was washed three times with detergent to remove excess ligands and finally baked on a 50 degrees hot plate for one minute. The thickness of the seven layers of the PbS QD film was 100 to 150 nm after the ligand exchange process. TBAI (10 mg/mL dissolved in methanol), CTAB (10 mg/mL dissolved in methanol), EDT (2% dissolved in acetonitrile), Na_2_S (10 mg/mL dissolved in formamide), the washing agents, were the corresponding solvents, with the difference being that for Na_2_S, the washing agents for the three washes were formamide, acetone, and isopropanol, respectively.

### 2.3. Characterization

The UV-Vis-NIR absorption spectra of PbS QDs were recorded using a UV-VIS-NIR spectrophotometer (HITACHI, U-3501, Tokyo, Japan). The sizes and separation distances of QDs were determined using transmission electron microscopy (TEM, FEI, Tecnai F20, Lincoln, NE, USA). The Fourier transform infrared absorptions (FTIR) spectra (Bruker, Vertex70v, Berlin, Germany) of the PbS QD films were measured to verify the as-synthesized ligands (OA) on PbS QDs were successfully exchanged. Field-emission scanning electron microscopy (SEM, Zeiss, Sigma300, Oberkochen, Germany) was also used to investigate the microstructures of thin films. Photodetector device performance was investigated with a semiconductor characterization system composed of a spectrometer (DSR-F4-XIAN, Zolix, Beijing, China), a precision source meter (Keysight B2901A, Santa Rosa, CA, USA), and a lock-in amplifier (Model SR830 DSP, Sunnyvale, CA, USA).

## 3. Results and Discussion

### 3.1. Microstructural Properties

**Figure 1:**
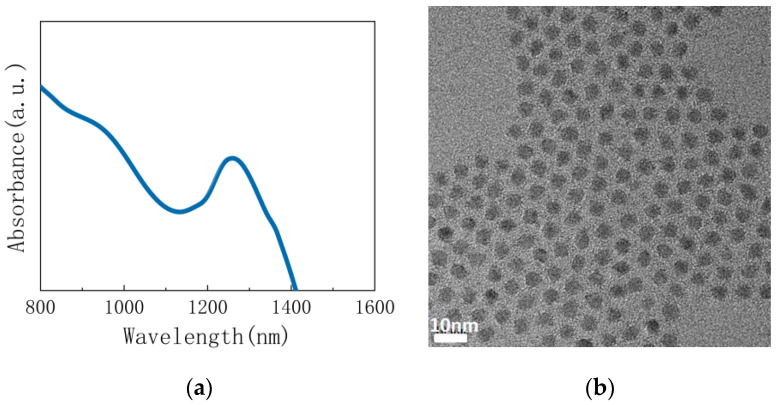
(**a**) Absorption spectrum of PbS QDs; (**b**) TEM image of PbS QDs in the presence of an oleic acid ligand.

**Figure 2:**
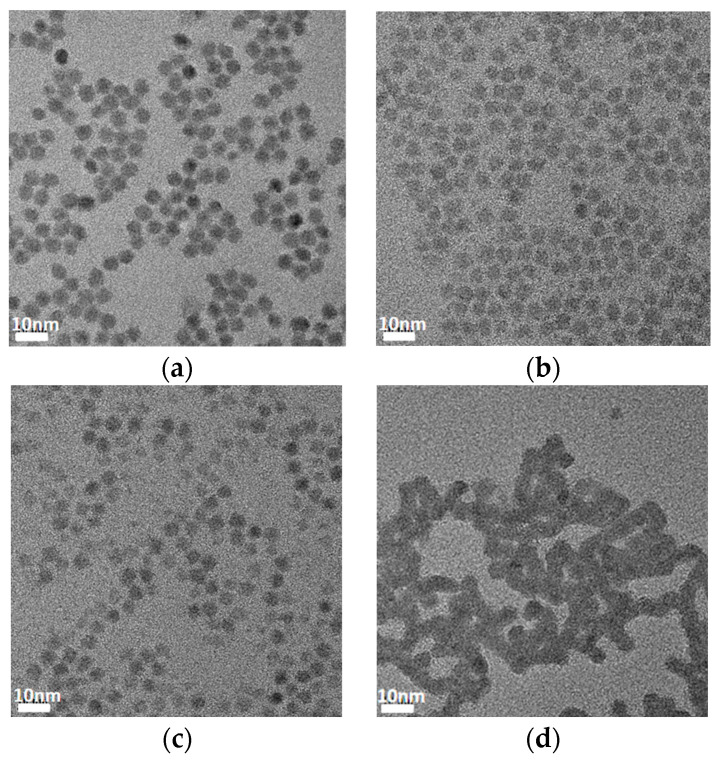
TEM images of the PbS QD films capped with (**a**) EDT, (**b**) TBAI, (**c**) CTAB, and (**d**) Na_2_S.

The absorption spectrum of the PbS quantum dots used in this experiment is shown in Figure 1a. A sharp exciton peak at 1220 nm was observed in the absorption (Abs) spectra of PbS QDs, indicating a low defect concentration of QDs and that the average particle size of the PbS quantum dots was approximately 4.6 nm. We fabricated TEM samples in order to compare the quantum dot spacing after treatment with ligands, and the results are shown in Figure 1b and Figure 2. The presence of ligands on the surface of QDs directly affects the conductivity of the films, and in QD films with oleic acid as the ligand, charge transport is suppressed, and insulating behavior is shown. Experimentally, it has been shown that, quantum dots have a relatively uniform particle size distribution. The distances between adjacent PbS quantum dots treated with ligands were reduced to approximately 0.5–1 nm, compared to the initial oleic acid ligand quantum dots, which were close to 2 nm [26]. The reduced nanoparticle spacing after ligand exchange further increased the proximity between nanoparticles, thereby enhancing charge transport [27]. For different types of ligands, the different crystal faces of the rock salt-structured lead sulphide nanocrystals differ in terms of spatial opportunity and affinity for binding to the ligand [28]. The Na_2_S-treated nanoparticles clearly reduced the inter-QD spacing and formed a fused QDs structure. Previous studies have shown that the use of sulphur salts to reduce the inter-particle distance of quantum dots resulted in the formation of molten nanocrystal structures with a sulphur shell layer [29]. 

**Figure 3:**
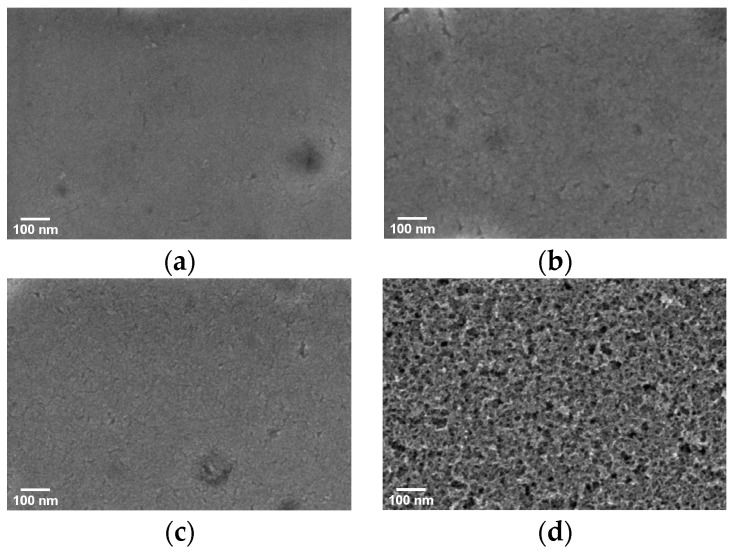
SEM images of the PbS QD films capped with (**a**) EDT, (**b**) TBAI, (**c**) CTAB, and (**d**) Na_2_S. (scale: 100 nm).

**Figure 4:**
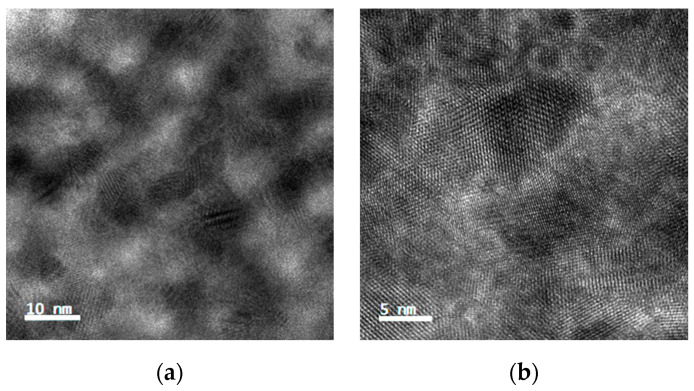
FIB-TEM images of PbS quantum dot films treated with Na_2_S at different scales. (**a**) 10 nm, (**b**) 5 nm.

Scanning electron microscopy (SEM) images of the PbS QD films (on silicon) after ligand exchange are shown in Figure 3a–d. From the images, ligand-treated quantum dot films can be found to exhibit defects, such as cracks or holes, which are detrimental to the transport of electrons in the films and may interfere with the performance of the optoelectronic devices, and are reduced by multiple spin coating [30]. There are considerable differences between the Na_2_S-treated quantum dot films and the other samples, exhibiting outstanding morphological characteristics. Further results using FIB-TEM, as shown in Figure 4a,b, indicate that the nanoparticles treated with Na_2_S are tightly aligned, and the adjacent quantum dots are connected to each other. There are clear lattice stripes and no obvious lattice defects in the quantum dots, which also demonstrates the high crystal quality of the synthesized PbS quantum dots.

### 3.2. Optical Properties

**Figure 5:**
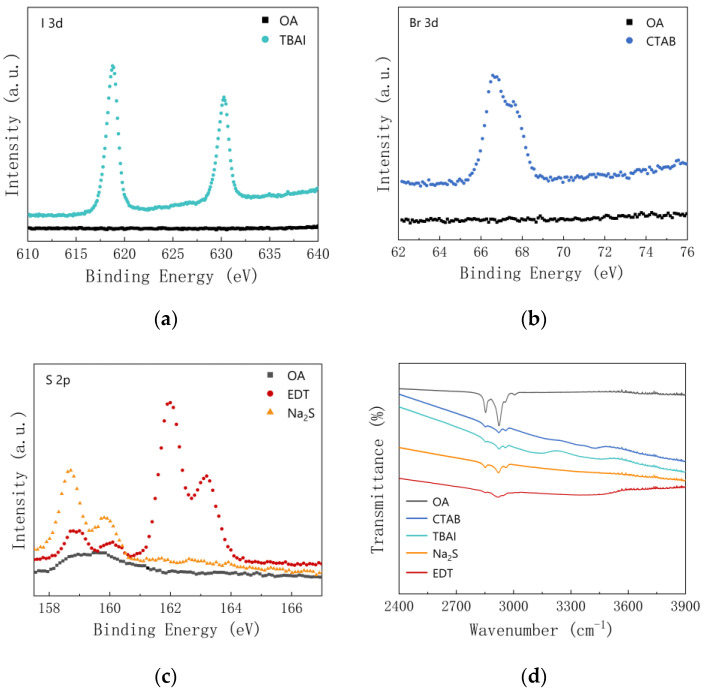
(**a**–**c**) XPS analysis of OA-treated (black), TBAI-treated (green), CTAB-treated (blue), EDT-treated (red), and Na_2_S-treated (orange) PbS QD films. (**d**) FTIR spectra of PbS QD films with different ligands. The spectra are offset for clarity.

We further used X-ray photoelectron spectroscopy (XPS) to examine the changes in the chemical composition of the PbS quantum dot films before and after the ligand treatment. Figure 5a shows Br3d scans of TBAI-treated PbS QD films, and Figure 5b shows I3d scans of CTAB-treated PbS QDs films, which indicate the presence of halide ligands (bromine and iodine) in the films after ligand exchange. The spectra in Figure 5c shows an increase in the amount of bound thio substitutes in the films after EDT and Na_2_S treatment. In the EDT-treated films, the Pb–SO_x_ peak near 162–164 eV acts as the dominant state for the presence of elemental sulphur on the surface of the quantum dots. The films before and after ligand exchange were characterized using FTIR spectroscopy, and the results can be seen in Figure 5d. In the processed films, the positions of the individual functional groups were essentially unchanged, but the change in intensity of the vibrational peaks was clearly indicative of a significant change in the profile of the ligand on the surface of the processed films. The amount of surfactant ligand present can be compared using the peak of the C-H stretching reaction, and the characteristic absorption peaks of the C-H bond at 2854, 2929, and 2961 cm^−1^ were all much weaker after ligand exchange as compared to the original oleic acid ligand, indicating that the great majority of the original long-chain oleic acid ligand was successfully substituted. Based on FTIR and XPS results, it can be concluded that the PbS quantum dots in the films were coated with halide ligands, as well as thiol and sulfur ions, after the solid-state ligand exchange process. PbS QD thin films prepared in an air environment generally showed P-type properties, for instance, with the addition of chalcogens from ligands, resulting in P-type QD films [29,31], while the PbS quantum dot films treated by TBAI showed N-type properties [32]. Some researchers use the PbS-EDT layer as the electronic blocking/hole extraction layer between the PbS-TBAI layer and the anode to improve the collection efficiency of photocurrent and device performance. The P-type semiconductor is used as the hole acceptor and transport layer, and the N-type semiconductor is used as the electron acceptor and transport layer. Due to the spatial separation of electrons and holes in two different mediums, effective charge separation at the nanoscale will inhibit the recombination of carriers [33].

### 3.3. The Device Application

We fabricated the photoconductive detector by spin-coating colloidal quantum dots in solution onto gold cross-finger electrodes, and the structure is shown schematically in Figure 5a. The device area was 6 × 4 mm^2^, and the effective photosensitive area was 0.39 mm^2^. For photodetectors, the responsivity (*R*) is an important parameter for assessing the photovoltaic performance [34]. It can be expressed as follows:

(1) $$R=\frac{I_{light}-I_{dark}}{P}=\frac{I_{photo}}{P}$$

In the equation, the currents of the device in light and dark are *I_light_* and *I_dark_*, respectively, the photocurrent is *I_photo_*, and *P* is the optical power irradiated to the device. In order to compare photodetectors with different types, the normalized detectivity (D*) is another key factor in evaluating the performance of photodetectors [35], which can be expressed by the following equation:

(2) $$D^{*}=\frac{R\sqrt{S}}{\sqrt{2qI_{dark}}}$$

where *R* refers to responsivity, *S* is the effective area under illumination, and *q* is the electron charge (1.6 × 10^−19^ C).

**Figure 6:**
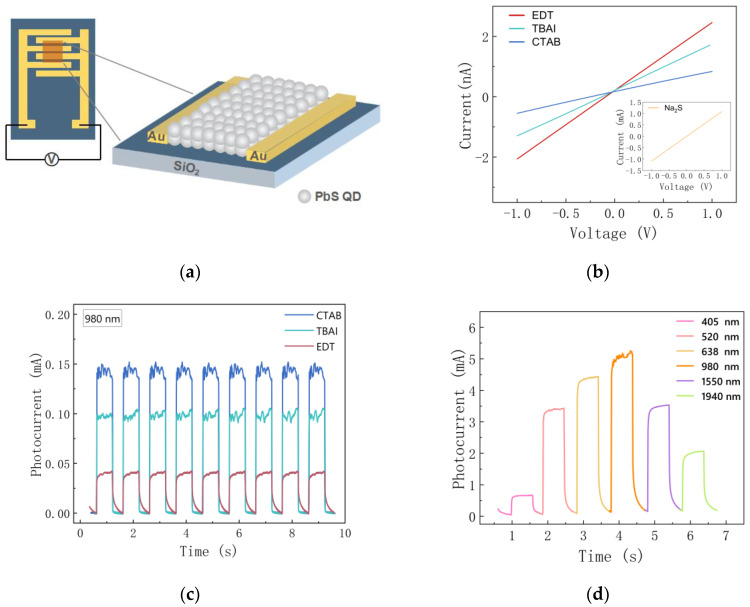
(**a**) Schematic diagram of the PbS QD photodetector structure. (**b**) The current–voltage (I–V) curve of the PbS quantum dot photoconductivity devices obtained in the dark. Insert of the figure shows the I–V curve of the PbS QDs device. (**c**) Photocurrent curves of the PbS-EDT/PbS-TBAI/PbS-CTAB photodetectors at 980 nm. (**d**) Photo response of PbS-Na_2_S photodetectors under incident light of 405 nm, 520 nm, 638 nm, 980 nm, 1550 nm, and 1940 nm (50 mW/cm^2^) at a bias of 1 V.

To investigate the optoelectronic properties of the devices, the current–voltage (I–V) curve of the PbS quantum dot photoconductance device was obtained under dark conditions, as shown in Figure 6b. The voltage was applied from −1 V to 1 V. The resistance value was estimated from the slope of the curve. As we can see, the resistance value of the device treated with sodium sulfide is too high, which is shown in the illustration. It can be seen that the resistance of the EDT-treated device is larger than that of the two halogen ligands with similar resistance, but both are far less than the PbS-Na_2_S device. Photocurrent curves of the devices were obtained under the light of a laser. The results of the PbS-Na_2_S photodetector tests were not in the same order of magnitude as those of the other three devices, so they were plotted in separate plots. Figure 6c shows the photocurrent curves of the PbS-EDT/PbS-TBAI/PbS-CTAB photodetector for irradiation at 980 nm with an applied bias voltage of 1 V. The dark currents for the EDT-, TBAI-, CTAB-, and Na_2_S-treated devices were 15.8 µA, 1.95 µA, 1.5 µA, and 2.2 mA. Figure 6d depicts the photo response detected by the PbS-Na_2_S photodetector, exhibiting a broad spectral response between 405 nm and 1940 nm. In this experiment, it can be seen that the PbS quantum dot photodevice achieves the maximum photocurrent under irradiation with 980 nm. The responsiveness and detection are calculated according to Equations (1) and (2), and the results are shown in Table 1. The responsivity performance of the device is ranked numerically as PbS-Na_2_S > PbS-CTAB > PbS-TBAI > PbS-EDT. The champion device achieved a response of 93.6 mA/W at 980 nm. The detectivities of PbS-EDT, PbS-TBAI, PbS-CTAB, and PbS-Na_2_S devices were 2.01 × 10^7^, 1.53 × 10^8^, 2.42 × 10^8^, and 2.28 × 10^8^ Jones, respectively. The PbS-Na_2_S photoconductive device not only had a higher order of magnitude of photocurrent than other devices but also a higher order of magnitude of dark current, due to the molecular structure of the linking groups and the stacking density of the nanocrystals, which can be more tightly packed together due to the Na_2_S treatment. This resulted in higher dark currents in the device, which also led to a lower specific detectivity. Previously, it was shown that dark currents have a significant effect on noise and specific detectivity [36,37]. 

**Figure 7:**
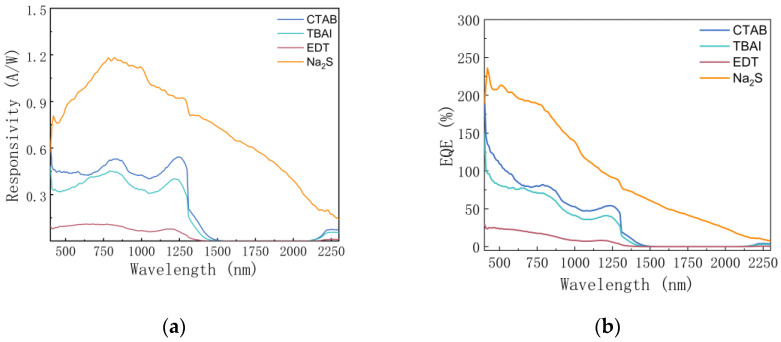
(**a**) The photoresponsivity (R) of the PbS QD photodetectors with the relationship of wavelength ranging from 400 nm to 2300 nm at V = 5 V; (**b**) external quantum efficiency (EQE) of the PbS QD photodetectors.

Figure 7a,b shows the spectral response and quantum efficiency of the PbS quantum dot devices treated with the four ligands. For The Na_2_S-treated PbS photodetector, the responsivity exceeded 0.9 A/W at around 1260 nm, and the quantum efficiency exceeded 95%, demonstrating a broadband photo response (400–2300 nm), indicating its suitability for visible and near-infrared light detection. It has been reported that Na_2_S-treated quantum dot films show high conductivity and mobility but rapid decay in photoconductivity, while EDT and TBAI show lower conductivity [38], which helps to elucidate the differences between photodetectors treated with Na_2_S and those treated with the other three ligands. 

We compared the performance of PbS photodetectors using various ligands in Table 2, and it can be seen that the spectral responsivity of PbS treated with Na_2_S in this paper was excellent. We attributed the low specific detection rate to the large dark current, which is also the limitation for photoconductive devices. It is worth reminding that most PbS quantum dot optoelectronic devices use composite materials or multilayer structures, which makes it difficult to explain the role of their ligands in device performance [15,39]. A direct comparison of devices with the same structure and different ligand-processing strategies helps to explain that the improvement of ligand strategy actually translates into excellent photoelectric detection performance, which is the significance of this work. 

**Table 2:** Summary of the performance of PbS quantum dot photodevices.

Type	Ligand	*R* (A/W)	D* (Jones)	Light Intensity	Reference
Photoconductor	Na_2_S	0.95	2.28 × 10^8^ (at 980 nm)	50 mW/cm^2^	This work
Photoconductor	EDT	--	3.3 × 10^11^ (at 1060 nm)	57 mW/cm^2^	[40]
Photodiode	TBAC	2.0 × 10^−13^	5.6 × 10^11^ (at 980 nm)	300 μW/cm^2^	[41]
Photodiode	TBAI	0.19	5.44 × 10^11^ (at 630 nm)	2.0 mW/cm^2^	[34]
Photoconductor	CTAB	9.0 × 10^−4^	8.9 × 10^9^ (at 630 nm)	430 μW/cm^2^	[42]
Photoconductor	MPA	--	3.9 × 10^9^ (at 630 nm)	430 μW/cm^2^	[42]

Ligand exchange affects the surface trap state of quantum dots, leading to a variation in decay kinetics and charge carrier lifetime with the type of ligand, as has been demonstrated in a number of studies [15,43]. This surface chemical treatment increases the carrier mobility of the nanocrystals and is accompanied by a reduction in the photogenerated carrier lifetime. In combination with TEM data, ligand exchange shortened the spacing of the quantum dots and even led to the fusion of adjacent particles in the sodium sulphide-treated quantum dots. While the enhanced interparticle coupling comes at the cost of larger excited-state lifetimes, the effect of the resulting high diffusion coefficients can be counteracted by the fast decay kinetics, leading to shorter diffusion lengths, which can lead to the rapid decay of the photoconductor [38]. The necking phenomenon is common in quantum dot films covered by ultra-small inorganic ligands and could explain the redshift and widening observed in the study. Although the mechanism behind this is not clear, it does not prevent us from considering it a promising ligand exchange strategy.

## 4. Conclusions

We compared the effects of four ligands, including halogen, thiol, and metal sulfide, on PbS quantum dots. The results showed that ligand exchange can indeed effectively remove long chains from the surface of quantum dots, shorten the spacing between quantum dots, and change the chemical element ratio and surface morphology of the quantum dot films, which in turn affects the optoelectronic performance of the device. Our study proves that sulfide and halogen ligands perform better than the current mainstream EDT applications. In particular, the sodium sulphide ligand-treated quantum dot photoconductor device not only had a higher order of magnitude of photocurrent for optimal performance, it also broadened the detection range, providing a promising approach for making highly sensitive PbS quantum dot NIR photodetectors.

## Figures and Tables

**Table 1:** Summary of the performance of PbS quantum dot photodevices.

Device	*I_photo_* (μA)	*R* (mA/W)	D* (Jones)
PbS-EDT	36.1	0.72	2.01 × 10^7^
PbS-TBAI	95.9	1.94	1.53 × 10^8^
PbS-CTAB	141.5	2.83	2.42 × 10^8^
PbS-Na_2_S	4780	95.6	2.28 × 10^8^

## Data Availability

The data supporting the findings of this study are available by reasonable request to yangmei@st.xatu.edu.cn.
